# Contribution of cytoskeletal elements to the axonal mechanical properties

**DOI:** 10.1186/1754-1611-7-21

**Published:** 2013-09-04

**Authors:** Hui Ouyang, Eric Nauman, Riyi Shi

**Affiliations:** 1 Department of Basic Medical Sciences, Purdue University, West Lafayette IN 47907, USA; 2 Weldon School of Biomedical Engineering, Purdue University, West Lafayette IN 47907, USA; 3 School of Mechanical Engineering, Purdue University, West Lafayette IN 47907, USA

**Keywords:** Axon, Elastic modulus, Atomic force microscope, Cytoskeleton

## Abstract

**Background:**

Microtubules, microfilaments, and neurofilaments are cytoskeletal elements that affect cell morphology, cellular processes, and mechanical structures in neural cells. The objective of the current study was to investigate the contribution of each type of cytoskeletal element to the mechanical properties of axons of dorsal root and sympathetic ganglia cells in chick embryos.

**Results:**

Microtubules, microfilaments, and neurofilaments in axons were disrupted by nocodazole, cytochalasin D, and acrylamide, respectively, or a combination of the three. An atomic force microscope (AFM) was then used to compress the treated axons, and the resulting corresponding force-deformation information was analyzed to estimate the mechanical properties of axons that were partially or fully disrupted.

**Conclusion:**

We have found that the mechanical stiffness was most reduced in microtubules-disrupted-axons, followed by neurofilaments-disrupted- and microfilaments-disrupted-axons. This suggests that microtubules contribute the most of the mechanical stiffness to axons.

## Introduction

The cytoskeleton is responsible for the spatial organization of the contents of the cell. It connects the cell physically and biochemically to the external environment, and also produces coordinated forces that permit cell motility and shape change [1,2]. Furthermore, the cytoskeletal architecture allows stresses applied to the cell to be broadly redistributed. Polymerization or depolymerization of individual elements can influence the entire network reorganization and ultimately alter the overall mechanical properties of the cell. Specifically, depolymerization of microtubules has been linked to biochemical cascades that can change actin dynamics in various cell types [3-7]. In the central nervous system, damage to the cells and extracellular matrix is poorly understood. Elucidating the mechanisms of such injury requires quantitative evaluation of the structure-function relationships at the sub-cellular, cellular and tissue level length scales.

An axon is a distinctive extension from the neural cell body that can be a few hundred micrometers in length or much longer, depending on the type of neural cell and the size of the species. The main function of an axon is to send impulses from the cell body to the neural dendrites. The myelin sheath protects and insulates the majority of axon fibers by wrapping around the axon and helping to transmit nerve impulses faster. Moreover, the axon also has unique cytoskeletal elements that are critical for its functional integrity.

The cytoskeleton of mature neurons contains three main types of filament structures: microtubules, microfilaments, and neurofilaments. Microtubules are about 25 nm in diameter. They are straight, hollow cylinders consisting of a ring of 13 micro-filaments that are made of dimmers of alpha and beta tubulins. Changes of length occurs at the plus end where polymerization of tubulin dimmers lengthens the structure and depolymerization of tubulin dimmers shorterns the microtubule. Microfilaments are only 5 nm in diameter, about the same thickness as the cell membrane. They are found throughout the neuron, especially in the neuritis. Microfilaments consist of two thin strands which are polymers of the protein actin. Actin is one of the most common proteins in neurons and is believed to be responsible for changing cell shape. Microfilaments are closely related to the membrane and are constantly undergoing assembly and disassembly. Neurofilaments are about 10 nm in diameter. They appear in all cells as intermediate filaments. They are called neurofilaments only when they are in neurons. Neurofilaments have multiple subunits arranged in a chain structure. Each subunit has three protein strands woven together, and each strand contains a long chain of protein molecules that are coiled in a tight and springlike configuration.

The cytoskeletal elements are the major structural units in axon, playing a crucial role in locomotion, division, and intracellular transport in axons. These different types of cytoskeletal elements are able to reorganize through a polymerization-depolymerization process and through a series of molecular motor actions that generate forces and motion using chemical energy. They also maintain the integrity of the cell by contributing mechanical support to the axons. However, the contribution of each type of cytoskeletal element to the mechanical properties of axons remains unknown.

PC-12, a cell line obtained from a pheochromocytoma of the rat adrenal medullar [8], has been broadly used to study the mechanical properties of neural cells and the contribution of cytoskeletal elements in maintaining the cellular structure of the cells. The mechanical response of PC-12 neurites under tension has been measured using a microneedle technique [9,10]. Bernal and colleagues reported the elastic modulus of PC-12 neurite to be approximately 12 kPa. At the molecular level, neurofilaments have been found to be a critical determinant of the overall shape and architecture of PC-12 neurites [11].

Atomic force microscopy (AFM) was first developed to provide nanometer-scale resolution in imaging traditional hard engineering and biological materials. The combination of the highly sensitive optical lever, precise movements by the scanner, and the careful control of probe-sample interaction allow researchers to measure force exerted on the sample with extremely high resolution. For the past two decades, AFM has been widely used to examine the mechanical properties of various cell types [12-15].

In this work, dorsal root ganglia and sympathetic ganglia cells from chick embryo were chemically treated to disrupt microtubules, microfilaments, neurofilaments, or all three elements. An atomic force microscope (AFM) utilizing cantilevers with spherical tips was then used to compress the axons of these neural cells. From the corresponding force-deformation information, Hertz contact theory was used to estimate the elastic modulus of axons under different cytoskeleton-disrupted conditions. Therefore, from this study, the impact of each cytoskeletal element on mechanical stiffness of axons from primary cultures was elucidated for the first time.

## Methods

### Primary cell culture

Dorsal root and sympathetic ganglia cells were dissociated from 8- to 9-day-old chick embryos [16]. Neural cells were collected from chick embryo in this study because the cell dissociation technique has been well developed in our lab. Nodules on dorsal roots or chains of sympathetic ganglia were collected and placed in Puck’s saline, and the ganglia were teased apart carefully using fine pointed forceps. Then the ganglia were digested with 0.01% trypsin in Puck’s saline and centrifuged for 5 minutes. Pre-made neural cell culture medium was added to the cell pellet, and cell density was determined with a hemacytometer. The initial cell density of cell suspensions was approximately 180,000 cells/60-mm-petri-dish, allowing the neural cells to have healthy processes within 24 hours. Prior to dissociation, petri dishes were coated with polyornithine (0.5 mg/ml in Borate buffer, 61.5 mM, pH 8.3) over night, then laminin (10 μg/ml in HBSS) for 4 hours. Cell suspension was added to the coated petri dish and incubated at 37°C in 5% CO_2_ up to 36 to 48 hours before further experiments. Neural cell culture medium was made with F12 nutrient mixture solution, and supplemented with horse serum, penstrep, conalbumin, vitamin C, and insulin.

### Disrupting the cytoskeleton

The pharmacological agents chosen are known to be selective in disrupting the corresponding cytoskeletal elements. Nocodazole destabilizes microtubules by competing for free tubulin [17,18]. Cytochalasin D disrupts microfilaments by binding to the filaments themselves [3,19]. Acrylamide causes collapse of vimentin filaments of the neurofilaments (or intermediated filaments in non-neural cells) [20,21]. Concentrations of chemicals were chosen so that cytoskeletons were totally disabled in axons of neural cells [22]. Nocodazole (15 μM), which interferes with the polymerization of microtubules, cytochalasin D (25 μM), which inhibits microfilaments polymerization, and acrylamide (4 mM), which promotes the disassembly of neurofilaments, or a combination of three agents was added to cell culture at 37°C in 5% CO_2_ for 2 to 4 hours.

### Immunocytochemistry

Immunostaining was used to visualize the cytoskeletons in neural cells. Cells were fixed with 4% paraformaldehyde for 25 minutes, and then rinsed with phosphate buffer solution (PBS) 3 times. Cells were permeated with 0.1% Triton X for 5 minutes, and then rinsed with PBS 3 times. To visualize microfilaments, AlexaFluor (working dilution 1:100; Invitrogen, Carlsbad, CA) was added to the cells and incubated at 37°C in 5% CO_2_ for 30 minutes. To visualize microtubules, monoclonal anti-β tubulin clone tub 2.1 FITC conjugate (working dilution 1:100; Sigma, St. Louis, MO) was added to the cells and incubated at 37°C in 5% CO_2_ for 60 minutes. Immunostaining of neurofilaments involved two steps. First, rabbit anti-neurofilament 200 IgG Fraction of Antiserum (working dilution 1:150; Sigma, St. Louis, MO) was added to the cells and incubated at 37°C in 5% CO_2_ for 60 minutes. Second, after rinsing cells with PBS for 3 times, secondary antibody Alexa Fluor 488 goat anti-rabbit IgG (H + L) (highly crossabsorbed) (working dilution 1:180; Invitrogen, Carlsbad, CA) was added to the cells and incubated at 37°C. All cells were observed under a fluorescent microscope, the Nikon Diaphot 300 (Nikon Instruments, Melville, NY).

### Atomic force microscopy

**Figure 1:**
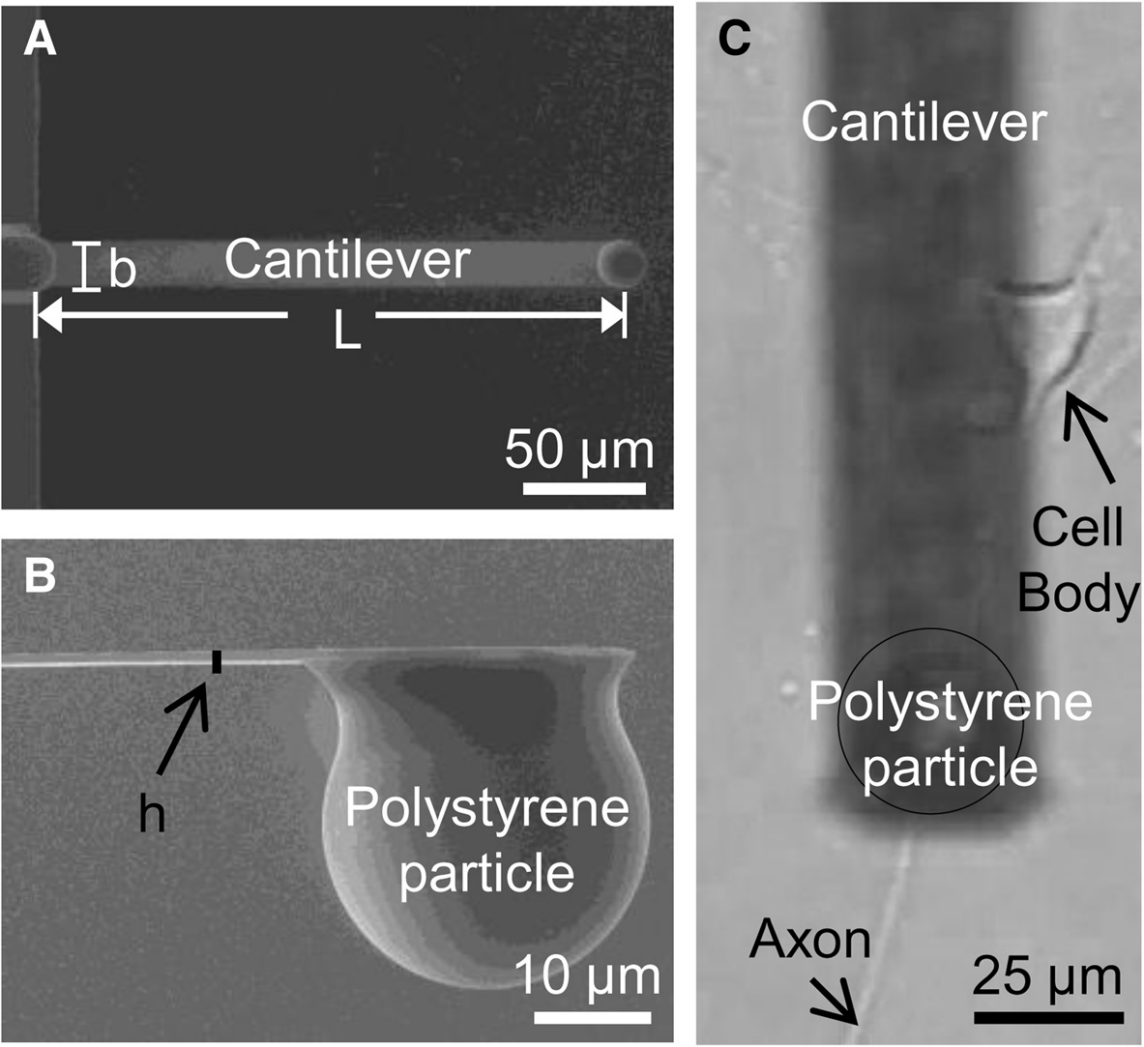
**Diagrams illustrating dimension of cantilever and axon being compressed by cantilever in atomic force microscopy (AFM). (A)** Top view of a cantilever obtained by scanning electric microscopy (SEM). **(B)** Side view of a cantilever tip obtained by SEM. **(C)** Top view of the particle of a cantilever compressing the axon of a dorsal root ganglia (DRG) cell. Image was taken by a digital camera from the eyepiece of the optical part of the AFM.

Force-deformation measurements on axons of neural cells were performed in neural medium using a BioScopeII AFM (Veeco Instruments, Plainview, NY) implemented with an Olympus IX71 optical microscope (Olympus Imaging America). All measurements were carried out at room temperature, 23°C. The cantilevers used in this study were PT.PS.SI (Novascan Technologies, Ames, IA) with elongated rectangular beams. A polystyrene particle with a diameter of 25 μm was attached to the end of the beam. The spring constant of each cantilever was calculated with a deflection equation that describes an end load on cantilever beam with single fixed support,

(1) $$P=\delta \left(\frac{3\;E\;I}{L^{3}}\right)$$

where *P* is force, δ is deflection of cantilever, *E* is elastic modulus of cantilever that is made of silicon nitride (*E*_*Si3N4*_ = 310 GPa), *I* is moment of inertia of cantilever based on its dimension, and *L* is the cantilever length. Thus, the spring constant (*SC*) of the cantilever is,

(2) $$\mathrm{SC}=\frac{3\;E\;I}{L^{3}}$$

and the moment of inertia, *I*, was determined by,

(3) $$I=\frac{b\;h^{3}}{12}$$

where b and h are the width and height of cantilever, respectively. The dimensions of the cantilevers were obtained with a scanning electron microscope (Figure 1A and 1B). The spring constants used in this study ranged from 0.056 to 0.56 N/m. Prior to the experiment, the sensitivities of each cantilever was calibrated in the neural cell culture medium contained within the petri dish by obtaining deflection curves on the dish surface. After the calibration, we located individual axons with the 40X objective of the optical microscope. The cantilever was engaged in contact mode with its tip positioned directly above the top aspect of the axon (Figure 1C). Once the cantilever tip touched the axon surface and stopped engaging, the AFM was immediately switched to force mode from scanning mode. The amount of compression on each axon was controlled by monitoring “Z-scan start”, a command that determines how far down the cantilever moves. The forward/reverse velocity of the cantilever was 4.08 μm/sec.

**Figure 2:**
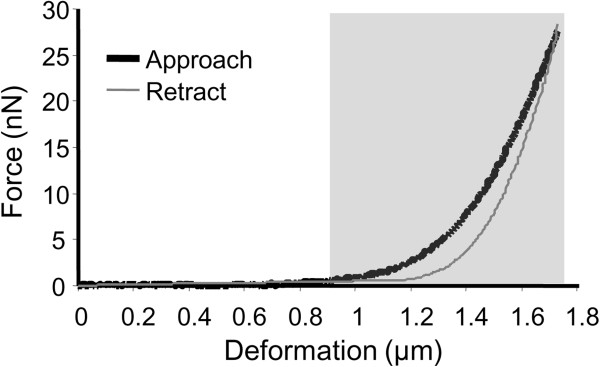
**Sample raw data of the force-deformation responses obtained from atomic force microscopy (AFM).** Only the approach curve was analyzed as it is the initial measurement of force responses in the compression test. The deformation at which the force amplitude was at least 2% of the maximum force was chosen to be the initial contact point between the cantilever and the axon (approximately 0.9 μm, gray area).

From the raw data of the force-deformation curves, only the first cycle of compression (Figure 2) was used because the maximum force amplitude decreased continuously with each cycle of the cantilever particle approaching and retracting from the axon (data not shown). In addition, only the approach curve was analyzed as the approach curve is the first force response in the compression test. In the each force-deformation curve, deformation referred to the actual distance that the cantilever traveled vertically as it was approaching the axon. In determining the initial contact point between the cantilever particle and the axon, the deformation at which the force amplitude was 2% of that at the maximum force amplitude was chosen. Therefore, in the example of the raw data of force-deformation curves (Figure 2), the tip of the cantilever contacted the axon at deformation of approximately 0.9 μm, and reached the maximum compression at deformation of approximately 1.72 μm. Hence, 0.82 μm (1.72 μm minors 0.9 μm) of the original thickness of the axon was compressed.

### Hertz contact theory

**Figure 3:**
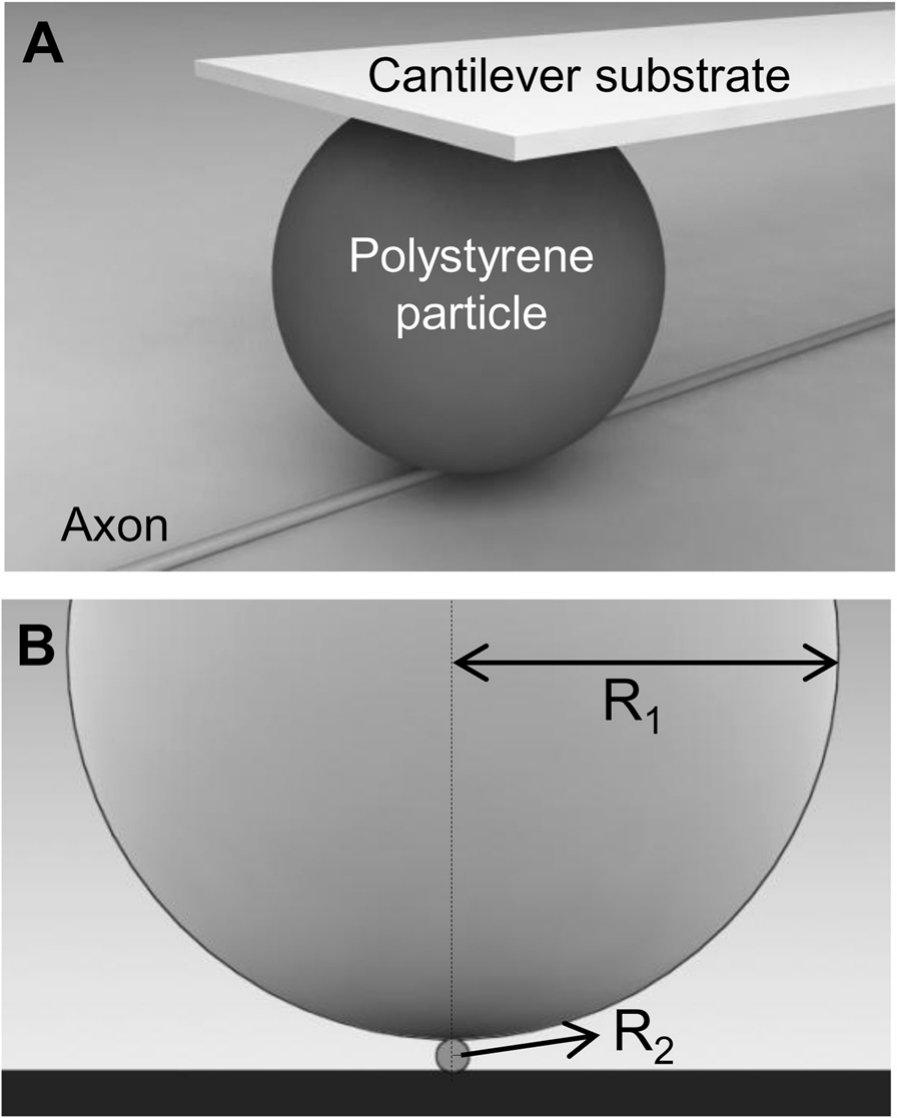
**Illustration of contact and dimensional ratio between the polystyrene particle and the axon in 3-dimension (A) and 2-dimension (B).** R_1_ is the radius of a polystyrene particle (12.5 μm), while R_2_ is the radius of the axon under compression (0.5 μm).

To provide an initial estimate of axonal mechanical properties, Hertz contact theory was employed. In this case, the two elastic bodies in contact were the hemispherical indenter (polystyrene particle), with radius, *R*_*1*_ = 12.5 μm and the cylindrical axon with an approximate radius, *R*_*2*_ = 0.5 μm (Figure 3), which generates an elliptical contact area [23,24]. The relative radii of curvature *R*΄_1_ and *R*΄_2_ and the effective radius, *R*_*e*_, are given by:

(4) $$R_{e}=\sqrt{R_{1}^{'}R_{2}^{'}}=R_{1}\sqrt{\frac{R_{2}}{R_{1}+R_{2}}}$$

It is also useful to define the effective modulus, *E*, for the two bodies in contact (Johnson, [24]),

(5) $$\frac{1}{E}=\frac{1-\nu_{1}^{2}}{E_{1}}+\frac{1-\nu_{2}^{2}}{E_{2}}$$

where the properties of the polystyrene particle are known (*E*_*1*_ = 3 GPa, ν_*1*_ = 0.34), and those of the axon were determined (Table 1). Hertz contact theory requires the applied force, *P*, to be related to the total displacement of the indenter-axon system, δ, through the following relationship,

(6) $$P=\frac{4}{3}E{\left(\frac{\delta R_{e}^{\frac{1}{3}}}{F_{2}}\right)}^{\frac{3}{2}}$$

where *F*_*2*_ is a correction factor based on the ratio of the effective radii of curvature (approximately 0.85 for this interaction (Johnson, [24]).

**Table 1:** Properties of polystyrene particle of AFM cantilever and axon used in calculation of Hertz contact theory

**Property**	**Polystyrene particle**	**Axon**
Radius	R_1_ = 12.5 μm	R_2_ = 0.5 μm
Poisson’s ratio	ν_1_ = 0.34	ν_2_ = 0.5
Elastic modulus	E_1_ = 3 GPa	E_2_ is unknown

Elastic modulus of axon, E_2_ was to be determined.

For each group of treated axons, the theoretical force-deformation relationship was compared to the averaged experimental data, and the value of *E* that minimized the total error between the two curves was determined. Assuming that the axon was comprised of an incompressible material, (Poisson’s ratio approximately 0.5), made it possible to solve for *E*_*2*_.

### Statistical analysis

One-way ANOVA was used to compare force amplitude at axonal deformation of 0.8 μm during compression by the AFM cantilever on normal axons to treated axons. Additionally, Tukey-Kramer multiple comparisons test was used as a post-hoc test to further analyze the differences among the normal and treated axons. P-values less than 0.05 were considered statistically significant. All data are presented in the form of means ± standard errors.

## Results

### Immunocytochemistry

**Figure 4:**
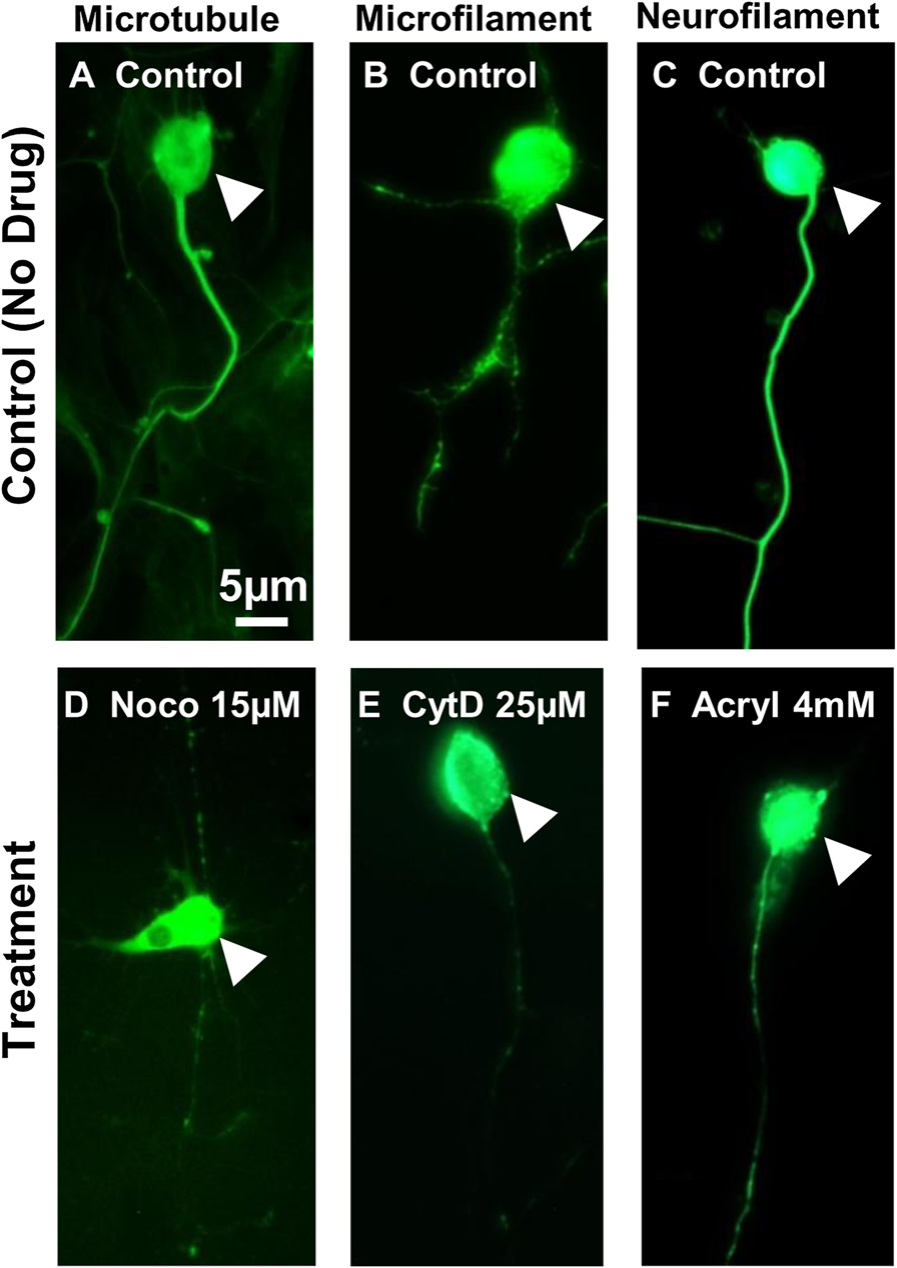
**Immunocytochemistry of dorsal root and sympathetic ganglia cells with disrupted cytoskeletal elements. (A**-**C)** Normal axons showed significant staining in microtubules, microfilaments, and neurofilaments. **(D**-**F)** Axons treated with 15 μM Nocodazole, 25 μM Cytochalasin D, and 4 mM Acrylamide showed significantly less staining in microtubules, microfilaments, and neurofilaments in axons, respectively. White arrows indicate cell bodies. Scale bar = 5 μm.

Normal dorsal root and sympathetic ganglia cells demonstrated obvious immunostaining of microtubule, microfilament, and neurofilament (Figure 4A-C). Cells treated with 15 μM nocodazole, 25 μM cytochalasin D, or 4 mM acrylamide disrupted microtubule, microfilament, or neurofilament, respectively, leading to significantly weakened immune-staining in each type of cytoskeletons (Figure 4D-F).

### Analysis of force-deformation data obtained from AFM

**Figure 5:**
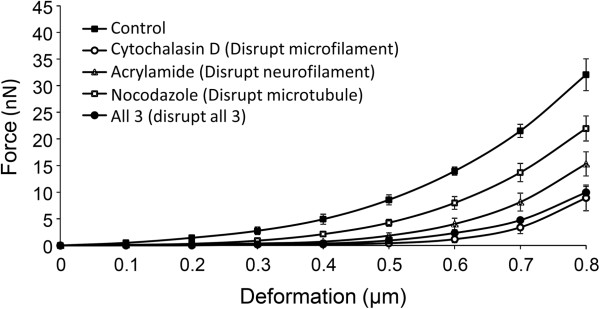
**Average force responses at each increment of deformation as the control and treated axons were compressed from 0 (no compression) to 0.8 μm.** Untreated control axons had the highest force response in each increment of deformation (n = 6), followed by cytochalasin D-treated (n = 6, microfilament disruption) and acrylamide treated axons (n = 6, neurofilament disruption). Nocodazole-treated axons (n = 6, microtubule disruption) and axons treated with all three drugs had similar force response in each increment of deformation.

Untreated control axons had the highest force response in each increment of deformation (average of 6 axons, Figure 5), followed by cytochalasin D-treated (average of 6 axons, microfilament disruption) and acrylamide treated axons (average of 6 axons, neurofilament disruption). Nocodazole-treated axons (average of 6 axons, microtubule disruption) and axons treated with all three drugs had similar force response in each increment of deformation. A one-way ANOVA demonstrated that untreated control axons had significantly higher force responses than any of the treated axons (Table 2) at maximum deformation (0.8 μm).

**Table 2:** Results of one-way ANOVA to compare forces at maximum deformation (0.8 μm) between control and drug-treated neural cells

**Treatment**	**Control**	**Disrupt microfilament**	**Disrupt neurofilament**	**Disrupt microtubule**	**Disrupt all 3**
Control	N/A	p < 0.05	p < 0.001	p < 0.001	p < 0.001
Disrupt microfilament	p < 0.05	N/A	ns	p < 0.01	p < 0.05
Disrupt neurofilament	p < 0.001	ns	N/A	ns	ns
Disrupt microtubule	p < 0.001	p < 0.01	ns	N/A	ns
Disrupt all 3	p < 0.001	p < 0.05	ns	ns	N/A

N/A: not applicable; ns: not significantly different.

### Determination of elastic modulus of axons

Elastic modulus of the axon under control and cytoskeleton-disrupted conditions was calculated by Hertz contact theory (Table 3). The average force amplitude at every 0.001 μm increment of deformation was calculated for all axons in each condition. The averaged force-deformation data (Figure 5) were used in the Hertz contact theory calculation. Under normal conditions (without drugs), the elastic modulus of the axon was determined to be 9,500 Pa. When microtubules were disrupted, the elastic modulus of the axon dropped to 1,470 Pa. The microfilament-disrupted axon had an elastic modulus of 5,785 Pa. The neurofilament-disrupted axon had an elastic modulus of 3,425 Pa. When all three cytoskeleton elements were disrupted, the axon elastic modulus was determined to be 2,020 Pa.

**Table 3:** Results of utilizing Hertz contact theory to calculate elastic modulus of axons with and without cytoskeleton disruption

**Drug**	**Disrupt cytoskeleton**	**Elastic modulus of axon, E**_**2 **_**(Pa)**
Control (no drug)	None	9,500
Nocodazole	Microtubule	1,470
Cytochalasin D	Microfilament	5,785
Acrylamide	Neurofilament	3,425
All 3 drugs	All 3 elements	2,010

## Discussion

**Figure 6:**
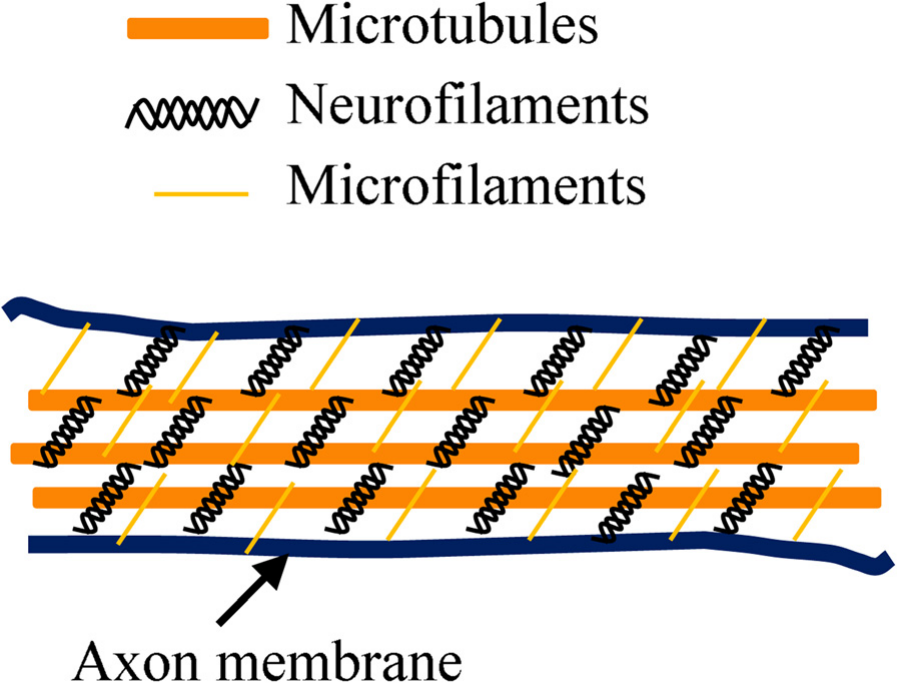
Proposed distribution of cyctoskeletal elements in an axon.

In this study, the elastic modulus of dorsal root and sympathetic ganglia cell axons from chick embryos was determined. AFM was used to perform compression on individual axons to obtain the force-deformation response. This information was evaluated with the Hertz contact theory to calculate the elastic modulus of the axons. To investigate the contribution of each cytoskeletal element (microtubule, microfilament, and neurofilament) to the stiffness of the axon, each element was disrupted, and the corresponding elastic modulus was calculated. Microtubule-disrupted axons had the lowest elastic modulus, suggesting that microtubules contribute the most to axonal stiffness. Microfilament- and neurofilament-disrupted axons also had lower elastic modulus than untreated axons. However, the effect was not as dramatic as with microtubules. Using the Hertz contact theory calculation, the elastic modulus of axons with all three elements disrupted was slightly higher than axons with solely microtubules disrupted. However, the difference may be insignificant given that there was no significant difference in force amplitude at maximum deformation between axons with microtubule depolymerization or all three element disruption. Such evidence suggests that microtubules not only provide the structure to support the mechanical stiffness of axons, but also offer a scaffold to support microfilaments and neurofilaments inside the axons. Once this scaffold is destroyed by microtubule disruption, the remaining two elements may not be able to maintain physical connections or integrity. Therefore, the contribution of the microfilaments and neurofilaments to the mechanical properties of axons is dependent on the presence of microtubules (Figure 6).

During the AFM experiment, three control mechanisms were performed to ensure the cantilever particle was compressing on top of the axon without shearing. First, the silicon nitride cantilever and the polystyrene particle are transparent under an optical microscope. Therefore, the axon was visible under the cantilever and particle. This helped the experimenter to ensure that the axon was under the middle of the particle before and during compression. This could be achieved by observing the motion through the eyepiece of the optical microscope. The limitation of this approach is that the resolution from the eyepiece of the optical microscope is on the micron-level, while the contact between the cantilever particle and the axon is in the nano-scale. While it is difficult for the user to position the particle exactly over the center of the axon, using a large cantilever particle increases the contact area with the axon surface, and minimizes error in the force-deflection curve. Second, measurements on axons slipping under the particle during compression were not considered. Only measurements on axons that remained attached to the petri dish surface during compression were used for data analysis. Third, the force amplitude when the cantilever particle was pressing against the petri dish surface was much larger than that generated by indentation on the axons because the petri dish surface is much harder than axons. Therefore, the experimenter was able to decide whether the cantilever particle was pressing against a hard dish surface or axons by observing the force responses during compression.

Hertz contact theory provides only an estimate of the elastic properties of the axon, but can be used to compare the effects of disrupting the cytoskeletal elements, but it should be noted that a more complete model is required to evaluate the injury process. In particular, the separate contributions of the cell membrane and large strain theories should be employed in order to establish the physical mechanisms that result in conduction deficits subsequent to mechanical insults. Previous work has examined the modeling required to obtain better estimates of cellular mechanical properties. Raman et al. [25] used a vibrational AFM system while Lin et al. [26] used a computational simulation of a spherical indenter pressing into a thick slab and compared their results to AFM-based measurments. The challenges with implementing both of these approaches is that the axons are approximately cylindrical in cross section. Consequently, performing vibrational testing or modeling the sphere-cylinder interactions were beyond the scope of the present study. For the purposes of this study, however, the relative contribution of each cytoskeletal element to the mechanical stiffness of axon was of primary interest. Therefore, Hertz contact theory was appropriate for this experimental objective. These data indicate that a complete mechanical characterization must include the cytoskeleton and cell membrane. Previous results further suggest that there is mechanical separation between the axon and myelin, especially near the Node of Ranvier, further emphasizing the need for multiscale models of the central nervous system [27].

The elastic modulus of axons was found to be approximately 9.5 kPa in this study, which is similar in range to previous findings in PC-12 neurites [10]. However, the mechanical properties obtained from the current study may be a more accurate reflection of axonal mechanical properties since DRG and sympathetic ganglia cells were used. These primary cells are actual PNS neurons rather than PC-12 cells, which are a cell line.

## Competing interests

The authors declare that they have no competing interests.

## Authors’ contributions

EN and RS conceived and designed the experiments. OH performed experiments and drafted the manuscript. All authors read and approved the final manuscript.
